# Neonatal Screening for Glucose-6-Phosphate Dehydrogenase (G6PD) Gene Variants and Their Association With Hyperbilirubinemia and Phototherapy Needs

**DOI:** 10.7759/cureus.102459

**Published:** 2026-01-28

**Authors:** Ismail M Alwadani, Raghad Almuslim, Mohammad Almutairi, Tahseen Elwahsh

**Affiliations:** 1 Department of Neonatology, Johns Hopkins Aramco Healthcare, Dhahran, SAU; 2 Department of Pediatrics, Johns Hopkins Aramco Healthcare, Dhahran, SAU

**Keywords:** g6pd, gene variant, hyperbilirubinemia, neonate, phototherapy, saudi arabia

## Abstract

Background and objectives: Glucose-6-phosphate dehydrogenase (G6PD) deficiency is highly prevalent in the Middle East and is a recognized risk factor for neonatal hyperbilirubinemia. However, the clinical impact of specific G6PD gene variants on hyperbilirubinemia severity remains unclear. This study aimed to determine the prevalence of G6PD gene variants among neonates at Johns Hopkins Aramco Healthcare and to evaluate their association with hyperbilirubinemia severity and phototherapy requirements.

Methods: We conducted a retrospective cohort study of neonates diagnosed with G6PD deficiency between January 2021 and December 2023. Demographic, clinical, laboratory, and genetic data were collected from electronic medical records. G6PD variants were identified using newborn DNA screening. Associations with phototherapy requirement were assessed using chi-square and Mann-Whitney U tests. Univariate and multivariate logistic regression analyses were performed to identify independent predictors of phototherapy.

Results: Among 5,375 neonatal admissions, 572 (10.6%) neonates were diagnosed with G6PD deficiency, with a male predominance (66.6%). The c.563C>T (Mediterranean) variant was the most prevalent (93.5%). Phototherapy was required in 193 neonates (33.7%). In multivariate analysis, female sex was independently protective against phototherapy (adjusted odds ratio (AOR) = 0.239; p = 0.003), while a positive Coombs test (AOR = 8.668; p < 0.001) and the presence of two mutant G6PD gene copies (AOR = 3.890; p = 0.007) were significant independent predictors of phototherapy requirement. No significant association was observed between specific G6PD variants and the need for phototherapy.

Conclusion: G6PD deficiency was common in this cohort and was mainly associated with the c.563C>T mutation. A positive Coombs test and multiple gene copies were independent predictors of phototherapy, whereas specific G6PD variants were not associated with hyperbilirubinemia severity. These findings support the importance of early G6PD screening and vigilant monitoring to prevent severe neonatal hyperbilirubinemia.

## Introduction

Glucose-6-phosphate dehydrogenase (G6PD) is a cytoplasmic enzyme in human red blood cells (RBCs; erythrocytes) that plays a vital role in the pentose phosphate pathway [1]. This enzyme catalyzes the conversion of nicotinamide adenine dinucleotide phosphate (NADP) to its reduced form, nicotinamide adenine dinucleotide phosphate hydrogen (NADPH). NADPH subsequently regenerates reduced glutathione, which protects erythrocytes from reactive oxygen species [2]. In individuals with G6PD deficiency, decreased NADPH production reduces glutathione production, leaving erythrocytes vulnerable to oxidative damage, hemolysis, and RBC destruction [2].

G6PD deficiency is the most common enzymatic disorder globally, affecting more than 400 million individuals [3]. The prevalence of G6PD deficiency varies considerably across ethnic populations [4]. The highest frequencies occur in sub-Saharan Africa and Southeast Asia, where the reported prevalence rates vary from 1% to 30%. In contrast, the prevalence in Europe and Japan is significantly lower, ranging from 0% to 10% [4].

In Arab countries, the prevalence is similarly high, ranging from 2% to 31% [3]. G6PD deficiency is highly prevalent in Saudi Arabia, with the G6PD-Mediterranean variant being the most frequent [5]. A 10-year retrospective study in Saudi Arabia screened 48,889 children hospitalized for G6PD deficiency and reported a prevalence of 25%, with a higher rate among men (33.8%) [6].

G6PD deficiency is an X-linked recessive enzymatic disorder; therefore, men are more likely to be affected than women [7]. Mutations in G6PD lead to protein variants that reduce enzyme activity and are linked to various biochemical and clinical phenotypes [8]. G6PD is located on the X chromosome (Strand Xq28), consists of 13 exons and 12 introns, and encodes a 515-amino acid protein with a GC-rich promoter region (>70%) [8].

Hundreds of biochemical variants, including more than 230 molecular polymorphisms of the G6PD gene, have been identified over the past 36 years, many of which occur in different populations [9].

A deficiency in G6PD reduces the ability of RBCs to withstand oxidative stress, leading to neonatal jaundice and hemolysis, which may also be triggered by fava bean ingestion and chemical agents [2]. Neonatal jaundice, which manifests as yellowish discoloration of the skin and eyes, is a common finding in newborns and indicates high bilirubin levels (hyperbilirubinemia) [10]. Hyperbilirubinemia occurs when the breakdown of RBCs exceeds normal levels, leading to the accumulation of bilirubin in the blood [10]. Other factors contributing to hyperbilirubinemia include impaired hepatic conjugation and enhanced enterohepatic circulation, as newborns exhibit elevated bilirubin levels due to a shortened lifespan of fetal hemoglobin, high hemoglobin concentration, and immature liver function [11]. In addition to the previously described causes, G6PD-deficient neonates experience a further reduction in RBC lifespan due to reduced enzyme activity, which is essential for maintaining cellular homeostasis and membrane integrity [7]. Furthermore, the presence of a uridine diphosphate glucuronosyltransferase 1A1 (UGT1A1) gene variant alongside G6PD deficiency has been recognized as a risk factor for neonatal hyperbilirubinemia, primarily due to reduced UGT1A1 activity in liver cells, which decreases bilirubin conjugation [7].

Although hyperbilirubinemia is reversible with early diagnosis and treatment, it can cause life-threatening complications when excessive bilirubin crosses the blood-brain barrier, leading to brain toxicity, acute bilirubin encephalopathy, and progression to kernicterus, an irreversible kind of brain injury linked to sensorineural hearing loss, aberrant posturing, arching, seizures, and cerebral palsy [12].

Many studies have found that multiple factors can influence hyperbilirubinemia in neonates with G6PD deficiency, including the presence of certain gene variants and the degree of enzyme activity [13,14]. Although studies on G6PD variants have provided valuable insights into the prevalence of G6PD deficiency in Saudi Arabia and worldwide, few have explored how these genetic differences contribute to the development of complications such as hyperbilirubinemia [15-17].

This study aimed to address this gap by investigating the prevalence of G6PD deficiency among neonates at Johns Hopkins Aramco Healthcare and exploring the correlation between G6PD gene variants and the occurrence and severity of hyperbilirubinemia. Identifying the key genetic factors associated with this condition underscores the importance of early screening to improve clinical management and prevention strategies for affected individuals, particularly neonates at risk of severe jaundice and kernicterus.

## Materials and methods

This retrospective cohort study was conducted at Johns Hopkins Aramco Healthcare Center in Dhahran, Saudi Arabia. This study focused on neonates with G6PD deficiency, comparing those who required phototherapy with those who did not, and examining their gene variants.

Data were retrospectively collected from 2021 to 2023 using an electronic medical records system. All collected data were securely stored in electronic systems, protected by passwords, and accessed only by authorized personnel. Patient identities remained confidential throughout the study period. This study received a consent waiver from the Institutional Review Board of Johns Hopkins Aramco Healthcare Center, Dhahran, Saudi Arabia, under log-number: 24-28.

The dependent variables analyzed included gestational age, birth weight, sex, peak serum bilirubin level, neonatal blood group, maternal blood group, Coombs test, duration of phototherapy, and G6PD gene variants. The independent variable considered was G6PD level (deficient or normal).

All neonates who underwent G6PD testing in cord blood and newborn screening (G6PD DNA analysis) were included; neonates with normal G6PD levels, incomplete G6PD screening, and neonatal death before specimen collection for newborn metabolic screening were excluded. Among 5,375 neonatal admissions during the study period, 572 met our inclusion criteria.

During hospitalization, cord blood samples were collected at birth from all newborns for G6PD. Cord blood G6PD samples were analyzed using fluorescent spot testing. Dried samples were examined under a UV lamp; fluorescence indicates normal activity, whereas the absence of fluorescence suggests a significant G6PD deficiency. At 24 hours of life, newborn metabolic screening was performed. Newborn metabolic screening included G6PD testing by DNA analysis. G6PD DNA analysis was performed to test for four mutations known to be associated with G6PD deficiency: c.[202G>A;3764>G], c.563C>T, c. 1376G>T, and c.1388G>A. Polymerase chain reaction and probe-hybridization methodologies were applied. If a variant was identified, we determined whether it was present as one copy, two copies, or as one copy of each of two different variants.

The need for phototherapy was determined based on peak serum bilirubin levels obtained during the first week of life and plotted on the hyperbilirubinemia nomogram adapted from the American Academy of Pediatrics (2004 or 2009). Total serum bilirubin levels were monitored after initiation of phototherapy to adjust for treatment requirements and intensity as indicated by the nomogram.

Statistical analysis

Data analysis was conducted using Statistical Package for the Social Sciences version 29.0 (IBM Corp., Armonk, NY, USA). The nonnormal distribution of continuous variables (birth weight, gestational age, and peak bilirubin level) led to reporting medians and interquartile ranges (IQRs). Categorical variables are presented as frequencies and percentages. Associations between categorical variables and phototherapy requirements were analyzed using chi-square tests, and Mann-Whitney U tests were used for continuous variables. Univariate logistic regression was used to identify potential predictors of the need for phototherapy. Multivariate logistic regression was performed to determine independent predictors of phototherapy requirements. A p value of <0.05 was considered statistically significant for all comparisons.

__PRESENT

## Results

Among the 5,375 neonatal admissions from January 2021 to December 2023, 572 (10.6%) were identified as having a G6PD deficiency. The cohort (n = 572) showed a male predominance (381, 66.6%). The c.563C>T mutation was the most prevalent (535, 93.5%), followed by c.[202G>A; 376A>G] mutation (21, 3.7%), and combined mutations (16, 2.8%). Single-copy variants were the most common (422, 73.8%). Phototherapy was required in 193 (33.7%) neonates, with most receiving 24 hours or less of treatment (149, 77.2%).

Blood group incompatibilities were ABO (78, 13.6%) and Rh (19, 3.3%), with a positive Coombs test in 29 (5.2%) cases. Phototherapy readmission occurred in 24 (4.2%) cases, with 20 (83.3%) requiring treatment for 24 hours or less. The median birth weight was 3,105 g (IQR, 2,760-3,400), gestational age 39 weeks (IQR, 38-40), and peak bilirubin 9.5 mg/dL (IQR, 7.0-12.1) (Table 1).

**Table 1:** Baseline characteristics and clinical outcomes of neonates in the study Rh: rhesus factor; IQR: interquartile range

Parameter	Frequency, n (%)
Sex of the neonate	
Male	381 (66.6%)
Female	191 (33.4%)
ABO incompatibility	
No	494 (86.4%)
Yes	78 (13.6%)
Rh incompatibility	
No	553 (96.7%)
Yes	19 (3.3%)
Coombs test for baby	
Negative	532 (94.8%)
Positive	29 (5.2%)
Need for phototherapy	
No	379 (66.3%)
Yes	193 (33.7%)
Duration of phototherapy	
≤12 hours	46 (23.8%)
24 hours	103 (53.4%)
48 hours	23 (11.9%)
72 hours	18 (9.3%)
96 hours	2 (1.04%)
120 hours	1 (0.5%)
Readmission for phototherapy	
No	548 (95.8%)
Yes	24 (4.2%)
Duration of readmission	
12 hours	5 (20.8%)
24 hours	15 (62.5%)
48 hours	3 (12.5%)
72 hours	1 (4.2%)
Gene variant	
c.563C>T mutation	535 (93.5%)
c.[202G>A; 376A>G] mutation	21 (3.7%)
c.[202G>A; 376A>g] mutation and c.563C>T mutation	16 (2.8%)
Number of copies	
1 copy only	422 (73.8%)
1 copy each	16 (2.8%)
2 copies	134 (23.4%)
Birth weight (g), median (IQR)	3,105 (2,760-3,400)
Gestational age (weeks), median (IQR)	39 (38-40)
Peak bilirubin (mg/dL) during the first week of life, median (IQR)	9.5 (7.0-12.1)

The analysis revealed several significant associations between the clinical characteristics and phototherapy requirements. Sex differences were significant (p = 0.032), with male participants showing a higher phototherapy requirement (36.7%) than female participants (27.7%).

ABO incompatibility demonstrated a strong association with phototherapy requirement (p < 0.001), as 57.7% of neonates with ABO incompatibility required phototherapy compared with 30.0% of those without it. The most striking association was observed with Coombs test positivity (p < 0.001): 86.2% of Coombs-positive neonates required phototherapy compared with 31% of Coombs-negative neonates.

Regarding genetic characteristics, although the c.[202G>A; 376A>G] mutation showed a higher phototherapy requirement rate (42.9%) than c.563C>T (33.8%) and combined mutations (18.8%), the difference was not statistically significant (p = 0.303). Similarly, the number of gene copies did not significantly influence phototherapy needs (p = 0.368), with similar rates observed across single-copy (34.8%), double-copy (32.1%), and combined variants (18.8%). Patients with Rh incompatibility showed a marginally higher phototherapy requirement rate (42.1% vs. 33.5%); however, this difference was not statistically significant (p = 0.433) (Table 2).

**Table 2:** Association between clinical characteristics and phototherapy need (n = 572) A p value of <0.05 is considered statistically significant

Parameter	Need for phototherapy		p value
	No, n (%)	Yes, n (%)	
Sex of the neonate			
Male	241 (63.3%)	140 (36.7%)	0.032
Female	138 (72.3%)	53 (27.7%)	
ABO incompatibility			
No	346 (70%)	148 (30%)	<0.001
Yes	33 (42.3%)	45 (57.7%)	
Rh incompatibility			
No	368 (66.5%)	185 (33.5%)	0.433
Yes	11 (57.9%)	8 (42.1%)	
Coombs test for baby			
Negative	367 (69%)	165 (31%)	<0.001
Positive	4 (13.8%)	25 (86.2%)	
Gene variant			
c.563C>T mutation	354 (66.2%)	181 (33.8%)	0.303
c.[202G>A; 376A>G] mutation	12 (57.1%)	9 (42.9%)	
c.[202G>A; 376A>G] and c.563C>T mutation	13 (81.3%)	3 (18.8%)	
Number of copies			
1 copy	275 (65.2%)	147 (34.8%)	0.368
1 copy each	13 (81.3%)	3 (18.8%)	
2 copies	91 (67.9%)	43 (32.1%)	

Analysis of continuous variables revealed significant differences between neonates who required phototherapy and those who did not. Birth weight was significantly lower in the phototherapy group (median, 3,040 vs. 3,130 g; p = 0.002). Similarly, gestational age differed significantly (p < 0.001); although both groups had the same median of 39 weeks, the phototherapy group had a narrower IQR (37-40 vs. 38-40 weeks).

In univariate analysis, several factors were significantly associated with phototherapy requirement. Female sex showed a protective effect (odds ratio (OR) = 0.661, 95% CI, 0.453-0.966; p = 0.032). Both lower birth weight (p < 0.001) and gestational age (OR = 0.773, 95% CI, 0.698-0.857; p < 0.001) increased the likelihood of requiring phototherapy. ABO incompatibility (OR = 3.188, 95% CI, 1.956-5.197; p<0.001) and a positive Coombs test (OR =1 3.902, 95% CI, 4.762-40.583; p<0.001) were strong risk factors. Gene variants and Rh incompatibility were not significantly associated.

After adjusting for confounding factors, only four variables remained independently significant. Female sex maintained its protective effect with a stronger association (adjusted odds ratio (AOR) = 0.239, 95% CI, 0.094-0.606; p = 0.003). A positive Coombs test remained a strong predictor, although with a reduced effect size (AOR = 8.668, 95% CI, 2.624-28.628; p < 0.001). Notably, having two gene copies emerged as a significant factor (AOR = 3.890, 95% CI, 1.444-10.478; p = 0.007), an association not apparent in the univariate analysis. Birth weight, gestational age, and blood type incompatibility were not significant factors in the adjusted model (Table 3).

**Table 3:** Univariate and multivariate logistic regression analysis of factors associated with phototherapy requirement A p value of <0.05 is considered statistically significant ^*^Peak bilirubin levels are evaluated using hour-specific, risk-stratified, and gestational-age-based nomograms AOR: adjusted odds ratio

Parameter	Univariate analysis			Multivariate analysis		
	B	p value	AOR (95% CI)	B	p value	AOR (95% CI)
Sex of the neonate, female	-0.414	0.032	0.661 (0.453-0.966)	-1.432	0.003	0.239 (0.094-0.606)
Birth weight (g)	-0.001	<0.001	0.999 (0.999-1.000)	-0.001	0.084	0.999 (0.999-1.000)
Gestational age (weeks)	-0.257	<0.001	0.773 (0.698-0.857)	-0.109	0.182	0.897 (0.765-1.052)
ABO incompatibility, yes	1.159	<0.001	3.188 (1.956-5.197)	0.554	0.088	1.740 (0.921-3.288)
Rh incompatibility, yes	0.369	0.435	1.447 (0.572-3.658)	0.247	0.675	1.280 (0.403-4.063)
Coombs test, positive	2.632	<0.001	13.902 (4.762-40.583)	2.160	<0.001	8.668 (2.624-28.628)
Peak bilirubin (mg/dL)^*^	0.343	<0.001	1.420 (1.324-1.524)	0.362	<0.001	1.437 (1.328-1.555)
Gene variant, c.563C>T mutation (reference category)						
c.[202G>A; 376A>G] mutation	0.383	0.395	1.467 (0.607-3.546)	-0.290	0.637	0.749 (0.225-2.490)
c.[202G>A; 376A>g] mutation and c.563C>T mutation	-0.796	0.219	0.451 (0.127-1.604)	1.033	0.275	2.808 (0.440-17.925)
Number of copies, one (reference category)						
One each	-0.840	0.195	0.432 (0.121-1.539)	1.033	0.275	2.808 (0.440-17.925)
Two	-0.123	0.560	0.884 (0.584-1.338)	1.358	0.007	3.890 (1.444-10.478)

__PRESENT__PRESENT__PRESENT__PRESENT

__PRESENT__PRESENT__PRESENT

__PRESENT__PRESENT

## Discussion

The World Health Organization classifies G6PD variants into five categories based on enzymatic activity and clinical presentations. Indirect neonatal hyperbilirubinemia commonly affects approximately 60% of full-term and 80% of preterm newborns during the first three days of life [18]. Class I variants exhibit severe enzyme deficiency and are associated with chronic nonspherocytic hemolytic anemia [19]. Several countries have implemented neonatal screening programs for G6PD deficiency [20,21]. G6PD deficiency is the most prevalent inherited enzymatic disorder, affecting approximately 5% of the global population [4]. Its incidence is particularly high in Saudi Arabia, although rates vary across provinces. According to the Ministry of Health, the prevalence among men is 8.4% [15]. A national literature review reported a higher prevalence (4%-14%) of G6PD deficiency among jaundiced neonates, many of whom developed jaundice within the first five days of life and required phototherapy or exchange transfusions [14]. Genetic mutations in G6PD deficiency have been linked to clinical manifestations, including hemolysis, especially in neonates [15].

In this study, among 5,375 neonatal admissions, 572 cases (10.6%) were diagnosed with G6PD deficiency. The c.563C>T mutation (G6PD Mediterranean) was predominant, accounting for 93.5% of cases. This finding aligns with a local study conducted in Jeddah, Saudi Arabia, which reported an 89.1% prevalence of the same mutation [22]. Similarly, studies conducted in Al-Hassa and Al-Qatif in the Eastern Province of Saudi Arabia identified the c.563C>T variant as the most common, with prevalence rates of 45.9% and 36.5%, respectively [23].

International studies have also confirmed the clinical significance of the c.563C>T mutation. At Aga Khan University in Pakistan, a study involving 216 icteric male infants readmitted for phototherapy identified G6PD deficiency in 32 neonates with reduced enzyme activity, among whom the c.563C>T mutation was associated with an increased risk of early-onset moderate hyperbilirubinemia [14]. In Gaza, a study of 65 pediatric patients with hemolytic anemia identified c.563C>T, c.202A/c.376G (G6PD A-), and c.404C>T (G6PD Cairo) as the predominant variants [24]. Similarly, a study conducted at Bechir Hamza Children’s Hospital in Tunisia found that the c.202A/376G and c.563C>T alleles were most prevalent among 154 neonates with jaundice, accounting for 44.8% of cases [25].

A more severe enzyme deficiency is strongly correlated with a greater need for phototherapy in infants. The c. 1388G> A and c. 1376G> T variants, commonly found in individuals of Chinese ancestry, were significantly overrepresented in the phototherapy group [26]. This finding aligns with a study of Chinese infants in Taiwan, which reported a positive association between the c.1376G>T variant and the need for phototherapy in neonates [27]. In this study, the c.[202G>A; 376A>G] mutation was associated with a higher phototherapy rate (42.9%) compared with c.563C>T (33.8%) and combined mutations (18.8%), although the difference was not statistically significant (p = 0.303). The number of gene copies did not significantly affect phototherapy rates (p = 0.368), with similar rates observed among single (34.8%), double (32.1%), and combined variants (18.8%).

These findings underscore the need for region-specific screening and management strategies for G6PD deficiency to reduce neonatal hyperbilirubinemia and its associated complications.

G6PD deficiency is a major cause of indirect neonatal hyperbilirubinemia, with the Mediterranean variant (c.563C>T) being the most prevalent in Saudi Arabia. However, some variants (e.g., c.[202G>A; 376A>G]) may present a higher risk requiring phototherapy. Universal newborn screening for G6PD deficiency is recommended in regions with a high prevalence to mitigate complications.

The study has some limitations. This study may not fully represent the genetic diversity across Saudi Arabia because it uses a single-center design. The absence of long-term follow-up data limits the assessment of neurological outcomes in G6PD-deficient neonates. The small sample size for rare variants reduced statistical power in subgroup analyses. Moreover, this study does not investigate the relationship between G6PD activity and gene variants. Future multicenter studies should examine genotype-phenotype correlations and optimize treatment thresholds.

__PRESENT

## Conclusions

While genetic variants alone were not a significant predictor of phototherapy requirements in initial analyses, the presence of two gene copies emerged as an independent risk factor after adjusting for confounders. This study illuminates the multifaceted interplay among genetic, hematologic, and demographic factors in neonatal G6PD deficiency and its clinical management, advocating the need for targeted screening and personalized care strategies and considering both genetic and hematologic profiles in G6PD-deficient newborns to optimize outcomes for affected neonates.
